# Sudan's rheumatic fever and rheumatic heart disease guidelines: a simplified approach in an endemic country

**DOI:** 10.3389/fcvm.2024.1403131

**Published:** 2024-05-10

**Authors:** Khalid M. Ali Sulafa, Zein A. Karrar, Nawal Elkurdufani, Nazik Ibrahim

**Affiliations:** ^1^ University of Khartoum, Khartoum, Sudan; ^2^ Sudan Medical Specialization Board, Khartoum, Sudan; ^3^ Federal Ministry of Health and World Health Organization, Khartoum, Sudan

**Keywords:** Sudan, rheumatic fever, rheumatic heart disease, guidelines, updated

## Abstract

**Background:**

Rheumatic heart disease (RHD) is a preventable sequelae of group A beta hemolytic streptococcal infection leading to an immune reaction: acute rheumatic fever (ARF) and progressive heart valve dysfunction. RHD is the leading cause of acquired heart disease in children and young adults in Sudan and many low/middle-income countries. In 2018, the World Health Organization (WHO) issued a resolution for RHD mandating that each country adopt updated guidelines for ARF and RHD management. These current guidelines are mainly directed to primary healthcare workers.

**Methods:**

Sudan’s Federal Ministry of Health (FMOH) in collaboration with the WHO East Mediterranean Regional Office (EMRO) assembled a committee for updating RHD guidelines. We conducted a systematic literature search from 2000 to 2022 in National Institute of Health Database (PubMed) under the following titles: streptococcal pharyngitis, acute rheumatic fever, rheumatic heart disease, benzathine penicillin. Best available, evidence-based practices for diagnosis and management of ARF/RHD were selected and adapted to Sudan's situation. The guidelines were critically appraised by the committee then endorsed to the FMOH and WHO EMRO Noncommunicable Disease Departments in January 2023. This paper describes the updated guidelines.

**Results:**

Simplified algorithms are provided for diagnosis of bacterial pharyngitis including two clinical criteria: sore throat and the absence of viral symptoms in the target age group. A simplified algorithm for diagnosis and management of ARF is adopted using two levels of diagnosis: suspected case at primary level where penicillin prophylaxis is started and secondary/tertiary care where echocardiography is performed and diagnosis confirmed or excluded. Echocardiography screening is recognized as the standard method for early diagnosis of RHD; however, due to the anticipated limitations, its implementation was not adopted at this time. Streptococcal skin infection is included as a precursor of ARF and a detailed protocol for benzathine penicillin administration is described.

**Conclusion:**

The Sudan guidelines for ARF/RHD management were updated. Endorsement of these guidelines to FMOH and WHO EMRO is expected to improve control of RHD in the region.

## Introduction

Rheumatic heart disease (RHD) is a completely preventable, most common cause of acquired cardiac mortality and morbidity in young people in Sudan and other low/middle-income countries affecting over 38 million individuals worldwide (1).

**Figure 1:**
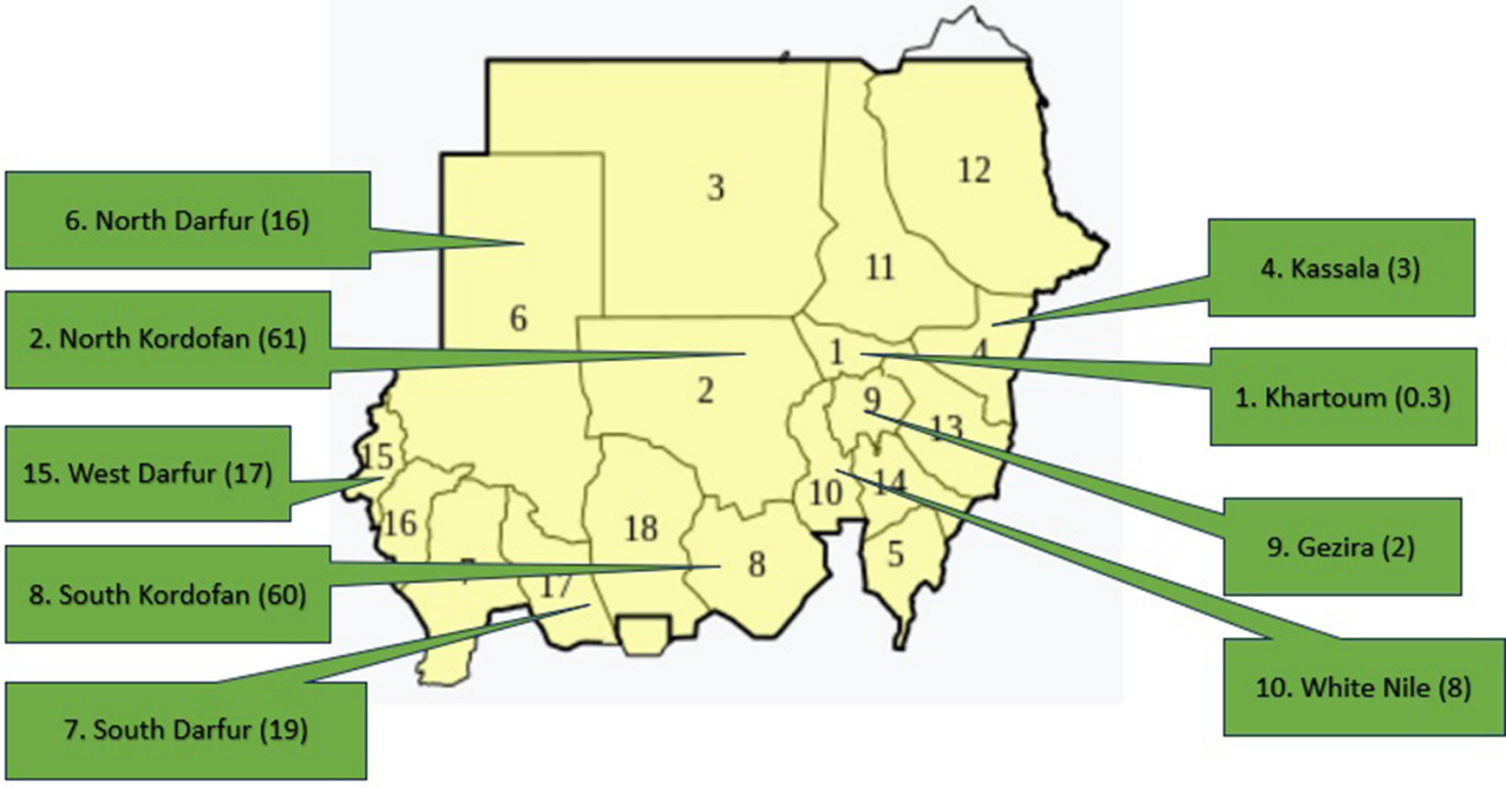
Sudan map showing the echocardiographic prevalence of RHD in nine states, numbers between brackets indicate the prevalence per 1,000.

Efforts to control RHD and its precursors streptococcal group A betahemolytic streptococcal (GAS) pharyngitis and acute rheumatic fever (ARF) need to be maximized to reduce the huge health and economic burdens of this disease. A World Health Organization (WHO)-based RHD control program was implemented in Sudan from 1986 to 1990. The program implemented clinical screening and early referral of school children in Khartoum State. The program reached its deadline in 2000 but was continued by voluntary efforts from the National Cardiac Center in Khartoum and continued to follow the WHO 2002 guidelines for RF/RHD until 2009 (2). A non-governmental program for RHD control was then established in 2012, and guidelines were produced for different levels of health workers with implementation projects in nine of Sudanese states (3, 4). The areas of high RHD burden were identified through a hospital registry and echocardiography (echo) screening (Figure 1). In referral hospitals, most patients were found to have established severe RHD and only 45% were compliant with benzathine penicillin G (BPG) prophylaxis (5).

In the current era, echo screening has been the standard method for early detection of RHD as its prevalence was found to be several folds higher than that detected by clinical auscultation (6). BPG was shown to stop the progression of echo-detected RHD, and this is expected to have important implications for RHD control as echo screening can potentially be incorporated into secondary prevention policies (7). Important modifications have been introduced to the methods of BPG administration, which have the potential to improve its uptake and decrease drug-related complications (8).

In 2018, WHO issued an RHD resolution that mandated that each country establish a control program for RHD (9). Consequently, a committee was assembled by the Sudanese Ministry of Health and the WHO East Mediterranean Regional Office in July 2022, aiming to update the Sudan ARF/RHD guidelines.

## Methodology

The guidelines were based on the previous 2017 published recommendations (4). We conducted a systematic literature search from January 2000 to July 2022 in National Institute of Health Database (PubMed) as well as published guidelines from recognized international organizations (Australia, New Zealand) under the following titles: streptococcal pharyngitis, acute rheumatic fever, rheumatic heart disease, and benzathine penicillin. Table 1 shows the classes and levels of evidence. The draft was written by first author and discussions and critical reviews were conducted by face-to-face and online discussions with the guideline committee members. The final version was endorsed in January 2023. The guidelines are directed to primary healthcare workers.

**Table 1:** Classes and levels of evidence.

Class	Definition
Class I	Evidence or general agreement that a treatment or procedure is beneficial, useful, effective
Class II	Conflicting evidence or divergence of opinions about usefulness
Class II-A	Weight of evidence in favor of usefulness
Class II-B	Usefulness not well established
Class III	Evidence or agreement that a treatment or procedure is not useful or may be harmful
Level of evidence A	Data derived from multiple randomized studies or meta-analyses
Level of evidence B	Data derived from a single randomized trial or large non-randomized studies
Level of evidence C	Consensus expert opinion or small studies

Color, Level of evidence; Green: Class I, Yellow, Class II-A, Orange: Class II-B, Red, Class III.

### Guidelines actionable recommendations

The main features of the updated guidelines are shown in Table 2.

**Table 2:** The main features of the updated guidelines.

Item	Previous Sudan guidelines (2017)	Updated guidelines (2022)	Rationale/evidence
ARF diagnosis	Modified Jones Criteria	Simplified criteria (arthritis, carditis, chorea)	- ARF could be transient - Evidence of high prevalence of subclinical carditis - Need to have a low threshold in highly endemic areas - Complexity of Jones Criteria Class I-B
ARF treatment	- BPG prophylaxis only after confirmation of diagnosis by Jones Criteria - Aspirin as anti-inflammatory	- BPG to all patients with arthritis, carditis, or chorea at primary care level - Ibuprofen as first-line treatment	- BPG can prevent progression of subclinical carditis - Ibuprofen is as effective and safer than aspirin Class I-B
Skin infection	Not included	Diagnosis and treatment of streptococcal skin infection is included as primary prevention of ARF	Evidence that skin infection can contribute to ARF Class II-B
Benzathine penicillin administration	No special precautions for patients with severe valve disease No sensitivity testing	- Not to be given for patients with severe uncontrolled heart failure - To give oral fluid before BPG injection - Graded oral challenge for patients with suspected mild allergy	Evidence that there are fatal non-allergic reactions to BPG in patients with severe heart failure Class II-A

Green: Class I, Yellow, Class II-A.

Orange: Class II-B.

#### 1. Diagnosis and management of bacterial pharyngitis (BP)

**Figure 2:**
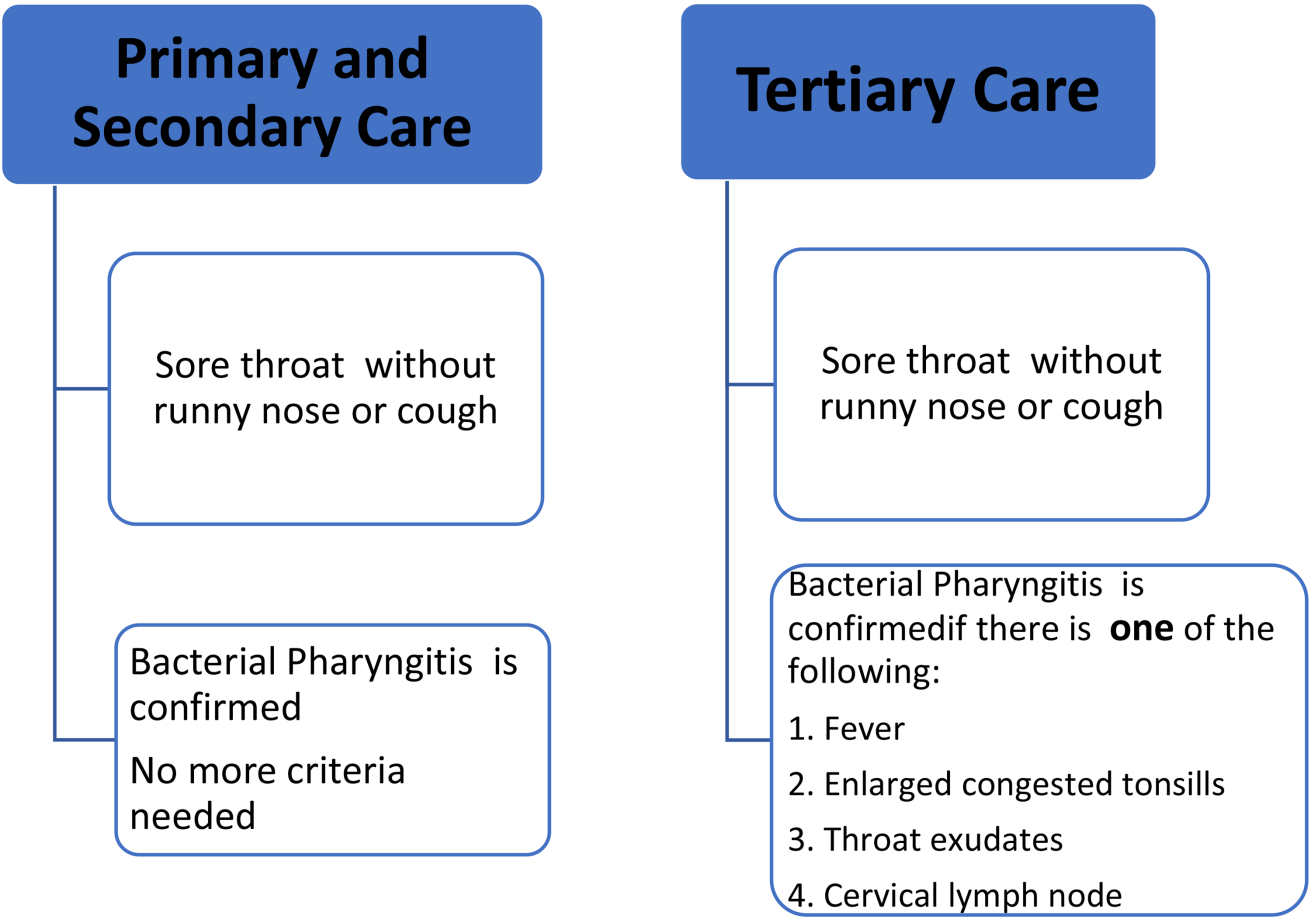
Diagnosis of bacterial pharyngitis.

In children aged 3–18 years living in RHD endemic areas, a clinical algorithm with high sensitivity is recommended for diagnosis of BP at primary healthcare settings (10–12). The diagnostic algorithm is shown in Figure 2. The first-line treatment is one injection of BPG (13–15). (For the dose and injection methods refer to the section on BPG administration). The second-line treatment is amoxicillin 40 mg/kg/day for every 12 h (max = 1,000 mg, same dose for adults) for 10 days. (For allergic patients, see the section on BPG allergy).

Patients and families need to be counseled about the importance of early treatment of pharyngitis as well as prevention by improving house ventilation and avoiding overcrowding and co-sleeping.

#### 2. Diagnosis and treatment of ARF

**Figure 3:**
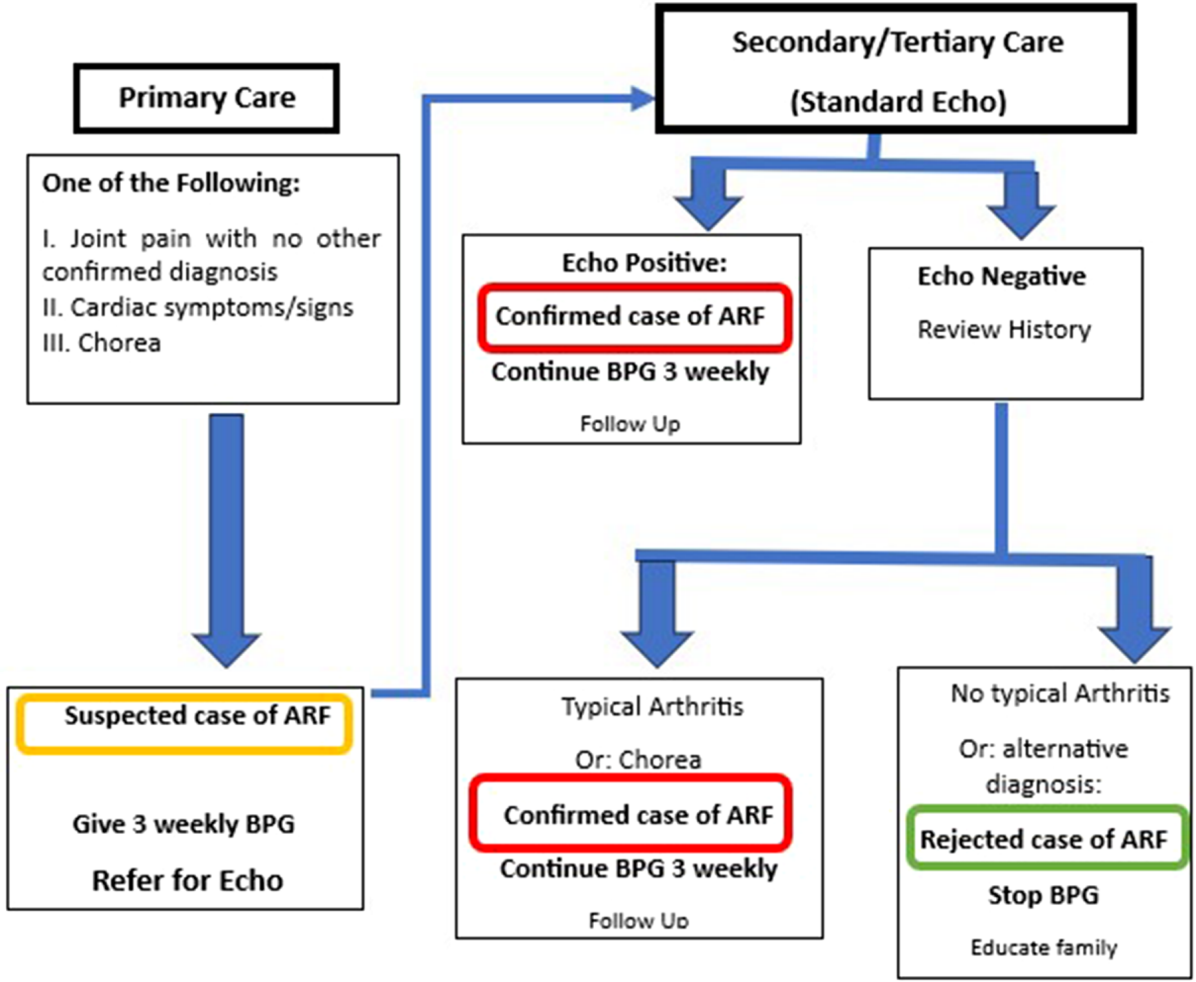
Diagnosis and management of acute rheumatic fever.

The algorithm for diagnosis and management of ARF is shown in Figure 3. This algorithm is simplified and modified from Jones’ Criteria, which has nine items (five major and four minor) and requires laboratory tests that might not be available in primary healthcare settings (16). All children with unexplained joint symptoms and heart murmurs indicating mitral and/or aortic valve disease need to be categorized as “suspected ARF.” In addition, those with chorea need to be classified as ARF unless there is an obvious alternative cause for this symptom. Standard ARF management including BPG prophylaxis should be started and referral to a higher center should be arranged. This simplified approach is expected to detect more cases of ARF in primary care settings and thus potentially improve prevention, early diagnosis, and treatment of RHD. (For the dose and injection methods, refer to the section on BPG administration). BPG should be continued every 3 weeks (for children below 18 years) and every 4 weeks for adults as secondary prophylaxis.

At the secondary/tertiary center, echo should be performed and the history reviewed to confirm or rule out ARF. If the patient presented to the secondary or tertiary care unit at the time of acute symptoms, then the standard Jones Criteria can be applied.

The duration of prophylaxis is up to 25 years of age if there is healed or no carditis and lifelong if there is persistent valve disease. If there is a contraindication for BPG, alternatives include oral penicillin V; the dose for those <27 kg is 250 mg twice/day and for those >27 kg is 500 mg twice/day for the duration of prophylaxis. (For allergic patients, see the section on BPG allergy).

Patients with ARF need to receive anti-inflammatory medications. The first-line treatment is ibuprofen 10 mg/kg/day every 8 h for 2 weeks. The second-line treatment is aspirin (60 mg/kg/day) every 8 h for 2 weeks. There is no evidence supporting the use of steroids; however, it can be used if non-steroidal anti-inflammatory medications are poorly tolerated. Prednisolone 2 mg/kg/day up to 80 mg/day for can be used for 2 weeks and then tapered and discontinued. Treatment of rheumatic chorea includes carbamazepine 5–10 mg/kg per dose twice daily or sodium valproate 5–10/kg per dose twice daily.

After resolution of chorea, drugs can be gradually discontinued (15). Consultation with a neurologist might be needed in refractory cases. Patients with heart failure should be started on diuretics: furosemide in oral or intravenous route in a dose of 1–3 mg/kg/day (children) and 40 mg every 8–12 h (adults) with spironolactone 1 mg/kg/day (children) and 25 mg orally every 12 h (adults). Consultation with a physician or cardiologist is advised for further management.

#### 3. Diagnosis and management of rheumatic heart disease

Echo was shown to detect RHD at an early (subclinical) stage in many countries including Sudan (17–19).

The World Heart Federation (WHF) recently updated echo criteria for RHD (20). The main updates include screening criteria for non-experts and then confirmatory criteria for experts. RHD was classified into five stages based on the risk of progression to more advanced disease. WHF recommended starting BPG prophylaxis for the earliest stage of subclinical disease.

Implementation of echo screening as a policy is not recommended in the current guidelines due to local country limitations; however, it can be used for studying the disease epidemiology and burden. Health workers need to be trained on the clinical diagnosis and management of RHD and its complications. Management and follow-up of patients with RHD are shown in Table 3.

**Table 3:** Management of patients with RHD.

Asymptomatic (clinical or sub clinical)	Symptomatic	Post-intervention
- Follow-up in tertiary care units - Ensure compliance with BPG - Dental hygiene and endocarditis prophylaxis - Pregnancy planning - Register and notify	- Treat heart failure - Follow-up in tertiary care unit - Ensure compliance with BPG for life - Comply with medications - Dental hygiene and endocarditis prophylaxis - Interventional treatment is planned by the cardiologist - Pregnancy planning - Register and notify	- Follow-up in tertiary care unit - Ensure compliance with anticoagulation medications and investigations - Ensure compliance with BPG for life - Dental hygiene and endocarditis prophylaxis - Pregnancy planning - Register and notify

Endocarditis prophylaxis should be considered for patients with RHD. Amoxicillin 50 mg/kg per dose for children and 2 g for adults can be used 1 h before procedures that lead to bacteremia. Patients need to be educated about the need to improve dental hygiene.

##### Post-intervention management

Patients with prosthetic valves need to continue anticoagulation (warfarin) for life. Warfarin dose needs to be adjusted according to the international normalized ratio (INR) as in Table 4 (21).

**Table 4:** Target INR for patients with prosthetic valves.

Prosthetic valve thrombogenicity	INR if *No* patient-related risk factors^a^	INR if more than 1 patient-related risk factors
Low^b^	2.5	3.0
Medium^c^	3.0	3.5
High^d^	3.5	4.0

^a^ Patient-related risk factors: mitral or tricuspid valve replacement; previous thromboembolism; atrial fibrillation; mitral stenosis of any degree; low left ventricular ejection fraction.

^b^ Carbomedics, Medtronic Hall, ATS, Medtronic Open-Pivot, St Jude Medical, On X, Sorin Bicarbon.

^c^ Other bileaflet valves with insufficient data.

^d^ Lillehei-Kaster, Omniscience, Starr-Edwards.

#### 4. Group A streptococcal skin infection

There is evidence that group A streptococcal skin infections (impetigo) alone or in combination with GAS pharyngitis may lead to ARF (22). Impetigo manifests as skin ulcers with honey-colored crusts on the face and extremities, which could be primary or secondarily infected insect bites or eczema. Treatment includes one injection of BPG (see the section on BPG administration) or cotrimoxazole (syrup 40 mg/5 ml, tablets 80/400 mg) twice daily for 3 days (15).

#### 5. Benzathine penicillin administration

BPG is the main drug used for primary and secondary prevention. BPG administration needs special training of health workers to improve both their uptake as well as and the compliance of patients (2). Important considerations when giving BPG are shown in Table 5.

**Table 5:** Important considerations when administering BPG.

Things that should be done	Things that should be avoided
Always ask about history of penicillin allergy	**Do not** give BPG to patients with history of **severe** allergy
Give 500 oral fluid before injection	**Do not** perform skin testing with dilute BPG
Let the patient lie for 5 min before injection	**Do not** give BPG to patients with uncontrolled heart failure or dehydration (23)
Use lidocaine 2% as diluent (24)	**Never** give BPG intravenous

##### Five-step protocol for BPG administration

**Step 1: Ask about allergy:**

• If there is no allergy: go to step 2.
• If there is a history of allergy, give an alternative medication: cefalexin 1 g (child: 25 mg/kg up to 1 g) orally every 12 h for 10 days for pharyngitis and erythromycin 250 mg twice per day for the duration of secondary prophylaxis.
• Refer the patient for confirmation of allergy if BPG is given for secondary prophylaxis (see management of allergy below).

**Figure 4:**
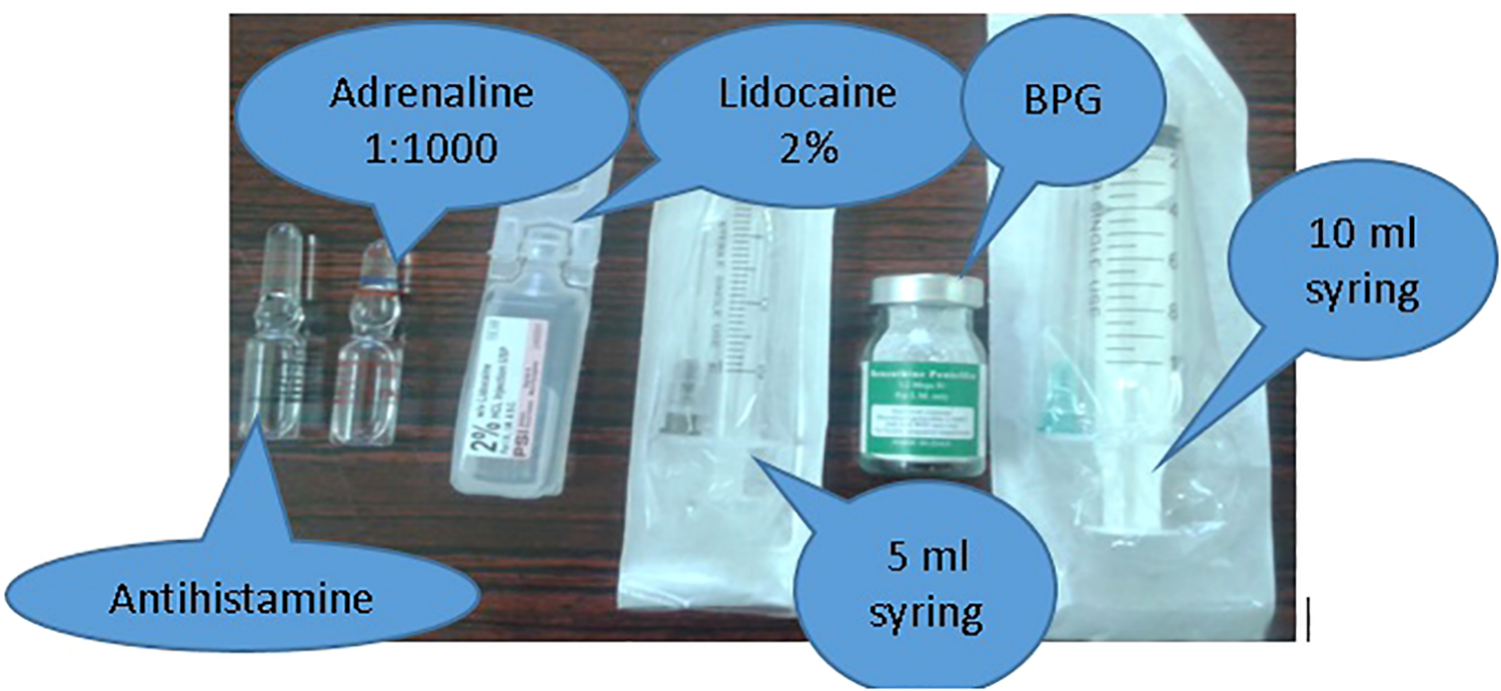
Items needed for BPG administration.

**Step 2: Prepare the items needed: shown in** **Figure 4**.

**Step 3: Aspirate lidocaine as a diluent and inject into the BPG vial.**

Using the 5 ml syringe needle, aspirate the volume of lidocaine denoted on the vial and inject into the BPG vial, shake till dissolved. Aspirate into the syringe.

**Warning for this step:**

IMPORTANT: TO INJECT BPG, CHANGE THE NEEDLE WITH A LARGE BORE (16 to 18 GAUGE) ONE (THE ONE PROVIDED WITH THE 10 ML SYRINGE)

BPG dose:

• For patients weighing 27 kg (about 9 years of age) or more: 1.2 million international units.
• For patients weighing <27 kg: 600,000 international units.

**Step 4: Prepare the patient and give the injection.**

• Ask the patient to take 500 ml of **oral fluid** as dehydration could precipitate vasovagal syncope.
• Let the patient **lie prone** for 5 min.
• **Using the large bore (16–18 gauge) needle**: insert the needle into the upper lateral quadrant of gluteus muscle. **Aspirate first** to ensure that you are not injecting into the vein. If **no blood** is aspirated: inject slowly.
• Document on patient records.

**Step 5: Observe for 15 min.**

• If the patient is well, discharge and **advise strict adherence** to doctor instructions.
• In case of mild allergy (itching), give antihistamine injection. In case of sever allergy/anaphylaxis follow the steps below.

Penicillin allergy:

Defined as a reaction to penicillin that appears shortly after the injection. There are two types of allergy:
  i. Mild allergy, which consists of skin rash, hives, itching, fever, and mild swelling.

Management of mild (skin only) allergy:

- If the reaction is seen, give oral or injectable antihistamine.
- In patients with ARF who experience mild allergy with a previous injection, an **oral graded challenge** can be given to test for allergy as follows (8):
  1. Give the patient 50 mg of oral amoxicillin syrup or one-tenth of a 500 mg crushed capsule.
  2. Wait for 30 min, if no symptoms give 450 mg of oral amoxicillin.
  3. Wait for another 30 min, if no symptoms proceed for the BPG injection.

ii. Severe allergy: manifests as airway tightness or anaphylaxis reaction characterized by:
  - tightening of the airways and throat, causing difficulty of breathing,
  - nausea or abdominal cramps,
  - vomiting or diarrhea,
  - dizziness or lightheadedness, and
  - low blood pressure leading to syncope and death.
  **Warning for this step:**
  DO NOT GIVE BPG TO PATIENTS WITH HISTORY OF THESE SEVERE SYMPTOMS

##### Management

• Maintain airway, Breathing and Circulation
• Call for help and act promptly to do the following steps:
• Intramuscular adrenalin 0.3 ml (<7 years) of 1:1,000 solution and 0.5 ml (>7 years), can be repeated in 15 min.
• Lie the patient with legs up
• O2 if the patient is distressed
• Intravenous normal saline and adrenalin infusion (25)

#### 6. Rheumatic heart disease and reproductive healthcare:

##### Risk stratification

Table 6 shows risk stratification of heart disease in pregnancy (26).

**Table 6:** Risk stratification of RHD in pregnancy (22).

Low risk (level I)	Elevated risk (level II)	High risk (level III)	Extremely high risk (level IV): pregnancy is contraindicated and termination considered
- History of ARF but no RHD - Mild asymptomatic RHD - Antenatal visit <20 weeks.	- Mild asymptomatic RHD with antenatal visit >20 weeks - Mild to moderate mitral or aortic regurgitation - Mild mitral stenosis - Bioprosthetic valve or previous mitral balloon commissurotomy - Mildly reduced ejection fraction (>45%) - Not level 3 or 4	- Mechanical valve - Moderate mitral stenosis: mitral valve are 1.5 cm–2 cm^2^ - Severe asymptomatic aortic stenosis - Severe asymptomatic mitral or aortic regurgitation - Moderately decreased ejection fraction (30%–45%)	- Severe symptomatic mitral stenosis: valve area <1 cm^2^ - Severe valve lesion with Pulmonary hypertension - Severe symptomatic aortic stenosis - Ejection fraction <30% - NYHA class III/IV

##### Preconception counseling

All adolescent girls should receive counseling about pregnancy and contraception according to their risk category including the following:

- Risk of pregnancy with RHD.
- Importance of planning pregnancy.
- Family planning options.
- Importance of visiting the specialist before getting pregnant.
- Patients need to have complete cardiac assessment (history, examination, and echo) for risk categorization before pregnancy.
- Patients who are considered high risk should be advised to take contraceptives **avoiding estrogens**. Intra-uterine contraceptive device and etonogestrel implant should be strongly encouraged (27).

##### Management during pregnancy and delivery

- Patients need to be evaluated by a **joint team** including obstetrics and cardiology. In high-risk patients, anesthetists and intensive care physicians need to be involved.
- Termination of pregnancy may be indicated if Level III or IV RHD severity is present.
- Secondary prophylaxis (BPG injections, oral penicillin, and erythromycin) are **safe** during pregnancy and breastfeeding, and should be continued.
- Angiotensin converting enzyme inhibitors, angiotensin receptor blockers, atenolol, and spironolactone should be avoided.
- Vaginal birth is associated with less blood loss, lower risk of infection, less venous thromboembolic complications, and is advised for most women with RHD.
- Post-delivery counseling includes the need to have cardiac evaluation, advice on contraception, cardiac medication, and secondary prophylaxis by specialist.
- Delivery: hemodynamic monitoring with supervision of anesthesia/critical care specialist, correction of anemia, and a short second stage are advised (26).

##### Anticoagulation

**Figure 5:**
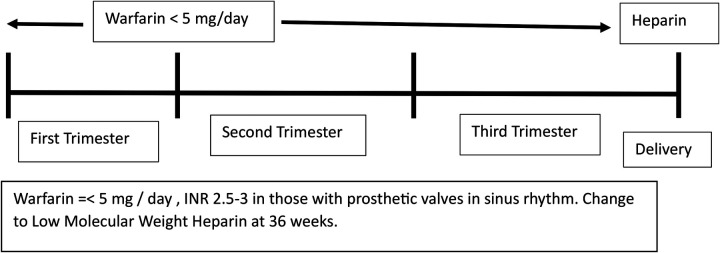
Management of anticoagulation during pregnancy.

- Warfarin is the drug of choice for patients with prosthetic valves but it can lead to fetal embryopathy.
- Direct oral anticoagulants such as rivaroxaban **cannot be used** for patients with prosthetic valves.
- Balancing maternal and fetal risks and individualizing the method of anticoagulation is best done by an **expert team**.
- The regimen of warfarin during pregnancy is depicted in Figure 5.

#### 7. Health system requirements for implementation of guidelines:

**Figure 6:**
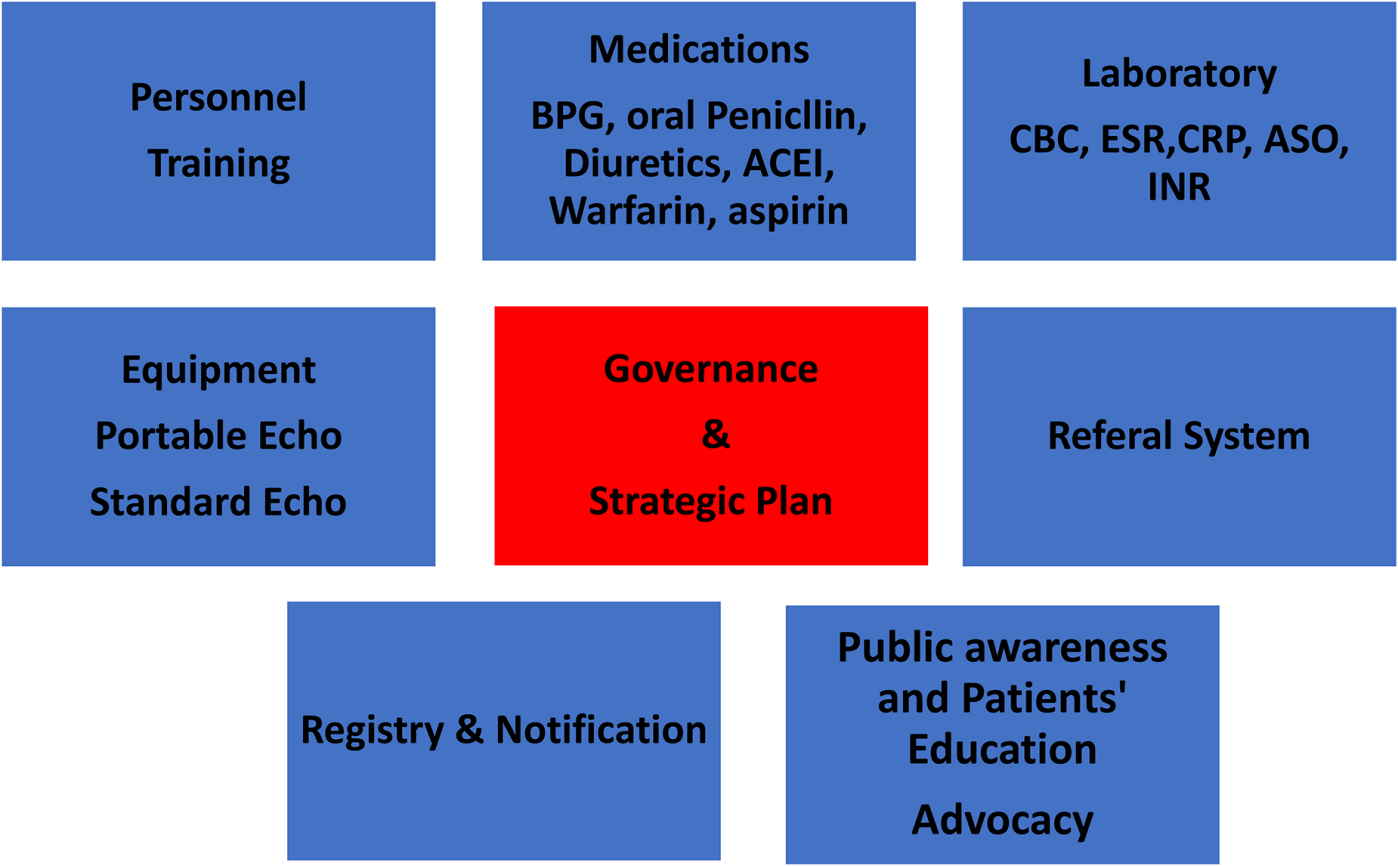
Health system requirements for implementing rheumatic heart disease guidelines.

Strengthening the health system especially at the primary and secondary levels is mandatory to control RHD. Patients with advanced RHD need tertiary care including surgery and cardiac catheterization, which poses a significant technical and financial burden on the health system. Figure 6 shows the items needed to implement the ARF/RHD guidelines at the primary and secondary care levels.

## Conclusion

Updated ARF/RHD guidelines were produced aiming to improve diagnosis and management of the disease at primary and secondary care levels. The guidelines were adapted to suit Sudan's health system facilities by utilizing simplified diagnostic and treatment methods. Implementation of these guidelines needs strengthening of the health system as well as establishing a governing body.

## Group members of RHD Guideline Committee

Abdel Moneim Elseed, Senior Consultant Pediatric Cardiologist and Professor at the University of Khartoum. Elfatih Abu Zeid, Senior Consultant Pediatric Cardiologist. Osama H. Elshazali, Consultant Pediatric Cardiologist at Ahmed Gasim Cardiac Center and Associate Professor at the University of Khartoum. Mohamed Elamin Ahmed. Consultant Pediatric Cardiologist at Wad Medani Cardiac Center and Associate Professor at Gazira University. Noha Eltag Karadawi, Consultant Pediatric Cardiologist at Ahmed Gasim Cardiac Center. Muawia E. A. Idris, Consultant Pediatrician at Omdurman Children's Hospital and Chief of Pediatrics Directorate at the Sudan Medical Counsel; Associate Professor, University of Bahri College of Medicine. Mohamed Awdelkrim A. Idris, Consultant Pediatrician at Ahmed Gasim Children's Hospital; President of Sudanese Association of Pediatricians. Osman Hamid Abdulhamid, Consultant Family Medicine and Rapporteur of the Counsel at the Sudan Medical Specialization Board; Assistant Professor at University of Gazira. Siddig Adam Ahmed, Consultant and Chair of Obstetrics and Gynecology Counsel at the Sudan Medical Specialization Board; Professor at the University of Khartoum. Magda Nogodalla, Consultant Public Health, Federal Ministry of Health, Noncommunicable Diseases. Amani Mohamed Abdelrazig, Federal Ministry of Health, Health Promotion Department. Samah Alfatih Alomda, Federal Ministry of Health, Noncommunicable Disease. Esmehan Alkheir, Federal Ministry of Health, Director of Mother and Child Health. Sara Elfatih, Medical Officer, Noncommunicable Disease, Federal Ministry of Health. Razan Abdulmajeed, Noncommunicable Disease Control Director, Khartoum Ministry of Health. Rayan Hayder E. Mahdi, Federal Ministry of Health, Clinical Pharmacist, Director-Rationale Use of Medicine. Ibtisam Faroug, Consultant Pediatrician, Ahmed Gasim Pediatric Hospital.
